# Anodal Stimulation - The Intrigue Continues

**Published:** 2011-05-01

**Authors:** Raja Selvaraj, Krishnakumar Nair

**Affiliations:** 1 Jawaharlal Institute of Postgraduate Medical Education and Research, Puducherry, India; 2 University Health Network, Toronto, Canada

**Keywords:** cardiac resynchronisation therapy, biventricular pacing, anodal stimulation

## Anodal Stimulation

When cardiac pacing is performed in the bipolar configuration, both the cathode (negatively charged electrode) and the anode (positively charged electrode) are in contact with the myocardium. Myocardial stimulation by the pacing stimulus typically occurs only at the interface with the cathode. This is because (i) given equal sized electrodes, anodal stimulation has a higher threshold compared to cathodal stimulation except for a "dip" at short coupling intervals [1] and (ii) the anode is usually designed as an electrode with a larger surface area making stimulation less likely. However, it has long been known that anodal stimulation is not uncommon with bipolar pacing systems because pacing output is usually programmed well above the stimulation threshold.

The effects of anodal stimulation, beneficial or otherwise, have been a subject of debate. Anodal stimulation was earlier on implicated as a cause of arrhythmias [2], especially in conditions of electrolyte imbalance or ischemia [3]. However, this arrhythmogenesis seems to be primarily related to pacing at a short coupling interval that could occur with older asynchronous pacemakers and may not be an issue with the current devices where pacing almost always occurs in a demand mode. On the positive side, anodal stimulation has been shown in animal models to improve conduction velocity [4] and mechanical performance [5] in the heart and for these reasons may even be considered desirable.

## Anodal stimulation in CRT

In modern CRT systems, bipolar LV pacing can be achieved by pacing between two LV electrodes (true bipolar) or from one of the electrodes on the LV lead to the RV ring (shared ring configuration). In this configuration it is known that anodal stimulation from the RV ring can be frequently demonstrated at higher outputs and may not be uncommon at usually programmed outputs [6]. This has been called "triple site pacing" as pacing occurs from the RV cathode, LV cathode and RV anode. The clinical significance of this phenomenon remains controversial.

On the one hand, anodal stimulation may have beneficial effects. As the term triple site pacing suggests, pacing occurs from three different foci and this may result in improved resynchronisation. Anodal stimulation results in a narrower QRS and improved resynchronisation demonstrated by tissue doppler imaging [6]. This was postulated to be due to activation of the inferior wall or septum by the ring electrode. However, it is recognised that three wavefronts may not always be generated by anodal stimulation. Depending on the interventricular timing delay and the proximity of the RV ring and tip electrodes, the wavefront from one of the RV electrodes may excite the myocardium adjacent to the second electrode, resulting essentially in two wavefronts only [7], so the benefit of improved resynchronisation may not always be present.

On the other hand, the effects of anodal stimulation may not always be desirable. In this issue of the journal, Dendy et al suggest that anodal stimulation may be a cause of clinical non-response to cardiac resynchronisation therapy [8]. The retrospective nature of the study, the diagnosis of anodal stimulation based only on the QRS morphology during LV pacing, the small number of patients and covariates that could have contributed to the lack of response make this an interesting hypothesis, but not a convincing conclusion. However, the results are strengthened in light of another recent study which suggests that a minority of patients with CRT may have worsening of LV function acutely with anodal stimulation while there was no change in most patients [9].

The mechanism of the deleterious effects of anodal stimulation is probably multifactorial. In a biventricular pacing system without anodal stimulation, there is separate activation of the LV lateral wall and the RV apex with the possibility to adjust the interval between these two. In the presence of anodal stimulation, we instead have activation at one or two sites in the RV along with the LV lateral wall. Interventricular timing delays are lost because anodal stimulation in the RV occurs at the same time as LV stimulation. Both the inability to preexcite the LV and the delivery of RV pacing from a location other than the apex could explain the negative consequences in some patients.

## Implications for the clinician

What should the clinician do in the face of this evidence? He must firstly be aware of anodal stimulation as a not uncommon phenomenon in patients with biventricular pacemakers. He must understand the factors that promote anodal stimulation, namely, programming LV-tip to RV-ring pacing configuration, use of a dedicated bipolar RV lead in defibrillators (instead of an integrated bipolar lead where the anode has a large surface area) and pacing at a high output. He must be able to identify anodal stimulation. The simplest way to do this is to look for a change in paced QRS morphology when output is gradually reduced with LV only pacing. Armed with the information that anodal stimulation may have variable positive or negative effects in individual patients, he can manipulate the output and pacing configuration in an attempt to improve the outcome in individual patients.

There is also a fascinating amount of research waiting to be done to unravel the role of anodal stimulation in response to CRT. CRT non-responders have to be prospectively evaulated for the presence of anodal stimulation to assess if this is more frequent than in responders. In non-responders with no other obvious cause for the lack of response, it must be studied if elimination of anodal stimulation can convert them into responders. At the same time, clinical applications for the beneficial effects of anodal stimulation found in experimental studies need to be probed. Exciting times lie ahead.
